# Supplementary material for: Dendrological Secrets of the Pazaislis Monastery in Central Lithuania: DNA Markers and Morphology Reveal Tilia × europaea L. Hybrids of an Impressive Age
Source: Plants (Basel). 2023 Oct 13;12(20):3567. doi: 10.3390/plants12203567 (PMC10610045; doi:10.3390/plants12203567)
Supplement: Supplementary file 1 [file plants-12-03567-s001.zip › plants-2611390-supplementary.pdf]

SUPPLEMENTARY MATERIAL

Table S1. Identification key of *Tilia platyphyllos*, *T.* x *europea*, and *T. cordata*

	Tilia platyphyllos -	*Tilia x europea*	*Tilia cordata*
Bark	Grey, grey-brown, longitudinally scarred [37].<br>Pale grey, in young trees smooth with longitudinal rows of shallow lenticels; these gradually become grooves that remain ill-defined and shallow until the trunk diameter reaches 0.25–0.30 m, when the grooves develop a rough, uneven surface and widen, separating longitudinal, sinuous ridges, 25–35 mm wide, horizontally cracked into rectangular scales that may loosen and detach[10].	Grey-brown [37].	Grey-brown, slightly longitudinally scarred [37].<br><br>pale grey, initially smooth with small lenticels that become longitudinal rows and eventually develop into narrow, shallow, longitudinal grooves between broad, smooth ridges (40–70 mm wide); at 50–70 years the ridges draw apart and become sinuous but remain linked by down widely directed, short, narrow ridges that divide the grooves into well-defined, diamond-shaped hollows [10].
Trunk	usually unbranched up to 15–18 m and the main branches are ascending or vertical to form an emergent. Often several-stemmed in woodland, either as a result of collapse and regrowth, or of cutting or coppicing; it may then form clonal groups of stems up to 10–20 macross. When growing in the open the trunk remains cylindrical but is short [10].	Tree with a cylindrical trunk, without bosses, sprouts sparse or absent;<br>°with massive woody bud-clusters or sprouts on the trunk **(T. × europaea var. europaea (= 'Pallida')) [10].**	cylindrical, unbranched to two-thirds of this height; lower branches of the first order horizontal and arching, upper branches ascending or vertical [10].
Crown	Wide, oval [37], parabolic or hemispherical, in open places a wide hemispherical or even conical crown [10].	Hemyspherical shape [10], broadly conical [37].<br>°Conical **(T. × europaea var. europaea (= 'Pallida')**) [10].	Egg-shaped, oval, 15-20 m in diameter [37], normally emergent, hemispherical, 5–12m in diameter [10].
Twigs	in the first year 2.0–3.8 mm in diameter, slightly flexuose, long-shoots glabrous, or with very sparse 3–4 limbed stellate hairs, shining, green, often becoming crimson in winter; short-shoots glabrous or with persistent hairs.<br>°Young twigs glabrous (***T. platyphyllos*** subsp. ***Pseudorubra).***<br>°Young twigs glabrous or sparsely hairy (***T. platyphyllos*** subsp. ***Platyphyllos)***	Hairy, later bare, reddish brown [37], thickness, Ø 2-4 mm [36].	Round, glabrous, reddish or yellowish brown, with light lenticels [37], bend at the buds [34], in their first year, slender, 1.3–3.5 mm in diameter, smooth, glabrous shining, olive green, green or crimson red, initially with sparse stellate hairs, which are normally lost, except on some short shoots [10].

	°Young twigs with simple and adpressed stellate hairs (***T. platyphyllos*** subsp. ***Cordifolia)*** [10].<br>Thick, reddish-brown, lilac shade, hairy, almost hairless in late autumn, with lenticels [37], reddish brown, hairy [34].		
Buds	7.0–9.5 mm long and 4.3–5.0 mm wide, ellipsoidal, or slightly ovoid, with three scales exposed, the lowest just over half the length of the bud, not swollen, glabrous [10], ovoid, 5-8 mm long, with an oblique base, pinkish red, glabrous or hairy with 3 scales, the outer one reaching half the length of the bud [37].	Buds usually with three scales exposed [10].	ovoid, 5.6–8.2 mm long and 3.6–5.3 mm wide, obtuse, with two scales visible (the hairy tip of a third may be visible at the apex), outer scale bulging, outer surface of both scales glabrous, shining, ciliate at the tip with two scale [10]s of unequal size [34]; broadly ovoid, obtuse, with an oblique base, repulsive, reddish brown, with 2-3 ciliated scales. The outer flagellum reaches more than half the length of the buds [37].
Petiole	2-5 cm long, hairy thin, 3-5 cm long, covered [37] with white bristly hairs [34]; 23–48 mm long, glabrous or with sparse stellate hairs; stipules 15–20mm long, 5–15mm wide, ovate, membranous with silky hairs on the **inner** surface [10].	3-5 cm long glabrous or slightly hairy [37]; petioles thick, clearly Ø 0.8-1.5 mm [36].	3-4 cm long, hairless, glabrous 1-6 cm long [37]; 21–46 mm long, slender, about 1.0 mm in diameter, glabrous or with sparse hairs; stipules 12–19mm long, 3–8mm wide, obovate, pale green, flushed red, translucent, with brown fasciculate hairs on the **adaxial** surface [10].
Leaves	Leaves are circular or slightly oblong, 6-15 cm long, sharply short-pointed, heart-shaped [37] or straight, often with an oblique base [34]; 59–109 mm long and 59–100 mm wide, orbicular or suborbicular,; acuminate, acumen 5–16 mm long, usually narrow; base varying between trees from almost symmetrically or asymmetrically cordate to obliquely truncate; margin entire at the base [10];°width predominantly 6-8 cm [36]..<br>Usually less than 60 mm in diameter (***T. platyphyllos*** subsp***. Corinthiaca***).	Generally, more than 65 mm in diameter, flat or weakly rugose, asymmetrically cordate at the base;<br>° obliquely truncate at the base **(T. × europaea var. europaea (= 'Pallida'))** [10].<br>Round or slightly elongated, thin [37]; leaflet often ovate or broadly ovate, width mostly ≤ length of midvein AND/OR secondary veins usually straight or slightly curved [36].	Round or oblong 5-7 cm long, pointed, heart-shaped base, sometimes cut slightly diagonally [37], curved sharply pointed apex [34]. 38–70mm long and 37–68 mm wide, orbicular or rarely suborbicular, acuminate with the acumen 5–15mmlong, narrow, usually straight; base slightly asymmetric, deeply to shallowly cordate, rarely truncate, flat or weakly rugose, dull [10].

	Usually more than 60 mm in diameter, normally (***T. platyphyllos*** subsp. ***Pseudorubra, T. platyphyllos*** subsp. ***Platyphyllos, T. platyphyllos*** subsp. ***Cordifolia)*** [10]; width predominantly >8 cm [34].		°Orbicular, more or less symmetrically cordate at the base and with a long acumen (***T. cordata*** subsp. ***Cordata)***<br>°Leaves suborbicular, cuneate or weakly cordate at the base ***(T. cordata*** subsp. ***Sibirica)*** [10].
Veins	With 6–9 pairs of main veins [10]; secondary veins 6-10/side [34].	The largest secondary vein with 6-7 branches ending in teeth; glabrous and/or hairy along the secondary veins, with forward hairs; Secondary veins 6-10 pairs [34].	With 4–6 pairs of main lateral veins, transverse veins evenly spaced, curved outwards, obscure in fresh leaves but, like the veins defining the islets, clearly visible when dry [10];<br>transverse vein lets rather irregularly branching, and poorly visible (10× LENS); secondary veins 4-6/side [36].
Leaves upper side	Dark green, without luster, covered with circles, short hairs [34] or almost glabrous [37]; dark green, usually slightly rugose, glabrous or with simple hairs along the main veins or sparsely on minor veins also;<br>°glabrous (***T. platyphyllos*** subsp. ***Pseudorubra, T. platyphyllos*** subsp. ***platyphyllos)*** [10] ***US glabrous (10× LENS).***<br>°US sparsely pubescent with single hairs (10× LENS). [36];<br>with sparse simple hairs ***(T. platyphyllos*** subsp. ***Cordifolia)*** [10].	Green, slightly hairy [37]	Smooth to slightly coarse, bare, dark green [10].
Leaves, lower surface	Dull green, covered with erect, white hairs [34]; green (not glaucous), glabrous or with sparse to dense, simple hairs, 400–700 μm long, perpendicular to the surface along the main and minor veins. Simple hairs may also form small, dense patches in the axils of the main veins, or they may be mixed with pale brown, fasciculate hairs, usually with only two arms [10]; moderately to densely pubescent along secondary veins with perpendicular hairs - vein axil pubescence disregarded (10× LENS) [36].<br>°T. platyphyllos subsp. corinthiaca some fasciculate hairs are normal;	Light green with tufts of whitish and yellowish-gray hairs in the branches of the veins [37]; large tufted hairs on branches of secondary veins, glabrous OR/IF pubescent along secondary veins, then with forward directed hairs - vein axil pubescence disregarded [36]; parallel, sharp veins of the third-row protruding to the surface of the leaf [34].	Pale green and glaucous, rarely mid-green, initially with scattered, stellate hairs but glabrous at maturity except for patches of brownish orange (rust-coloured), tangled, fasciculate hairs with 2–8 arms, up to 1 mm long, in the axils of main veins, extending over the veins at the base, along the veins and often as 4–6-armed stellate hairs on to adjacent parts of the lamina (these hairs are pale and sparser on shade leaves and may be entirely absent from the

	in other subspecies they may be evidence of hybridity, with small patches of simple and fasciculate hairs in axils of the main veins.<br>Without fasciculate hairs in the axils of the main veins:<br>Glabrous (***T. platyphyllos*** subsp. ***Pseudorubra)***<br>with sparse simple hairs along main and sometimes minor veins ***(T. platyphyllos*** subsp. ***Platyphyllos)***<br>sometimes confined to main veins, and sparse to relatively dense on the lower surface, particularly conspicuous along main veins (***T. platyphyllos*** subsp. ***Cordifolia)*** [10]; secondary veins with perpendicular hairs [36].		large leaves on vigorous sprouts from cut trees); stellate hairs otherwise absent.<br>° greyish (bluish) greenish, Tufted reddish hairs on the branches of the main veins; with small brownish spots, tufts of hairs are also present in the axillae of the secondary veins (T. cordata subsp. Cordata) [10];<br>The veins of the third row are very faint, crooked, and non-parallel [34].
Marginal teeth	Ciliate, saw-toothed, downward sloping [37]; teeth single or double, triangular, 2.5–3.6 mm at the base, usually with convex sides and prominent tip 0.4–1.0 mm long [10].	Semicircular to triangular with a blunt tip or with a short apiculus, less than 0.8 mm long [10]; teeth apiculi short, apiculi predominantly short and +/- blunt <0,8 mm (10× LENS). [36].	With both single and double teeth that are triangular with convex sides and directed slightly forwards, 2.6–3.6mm across the base, subacute but with a blunt pale green tip 0–0.4 mm long but normally without an apical extension of the vein [10]; serrated, edges curled up, finely serrated, sharply pointed, almost blunt [37].
Inflorescense	Peduncles 13–36mm long, glabrous; pedicels 7–17 mm long, slender, 0.5 mm in diameter, slightly thickened upwards, glabrous; inflorescence pendulous, often once-branched with 3 flowers, or more rarely twice-branched with 4–7 flowers [10]; cymes with 1-6 flowers [36]; with three, rarely 4-6 flowers [34].	Cymes pendulous at flowering, a normal dichasium; along the shoots [37];<br>Cymes 7 (–9) flowers, cyme with very short branches and long pedicels, almost an umbel (widely planted in northwest Europe (T. × europaea var. europaea (= „Pallida“)) [10].	Peduncles fused to the lower 0.45–0.64 of the bract, 8–23 mm long, slender, glabrous or with sparse hairs; pedicels 3–13 mm long, slightly thickened upwards; cymes with up to 5-30 flowers [10]; 3-11 flowers in an inflorescence [37]; 5-11 flowers [34];<br>Cymes usually withmore than five flowers (***T. cordata*** subsp. ***Cordata)***<br>Cymes with 3–5 flowers (***T. cordata*** subsp. ***Sibirica)*** [10].
Flowers	flowers with 25–45 stamens, large, 12–17 mm in diameter, saucer-shaped, petals often becoming slightly reflexed, sweetly scented. Sepals 5 mm long and 3 mm wide, ovate acute, shallowly	Bracts oblong or lanceolate. Cymes a normal dichasium<br>°Cymes 7 (–9) flowers, cyme with very short branches and long pedicels, almost an umbel	With 25–35 stamens, 12–15mmin diameter. Sepals 4mmlong and 2mm wide, almost elliptical, abaxial surface pale green, largely glabrous

	boat-shaped, outer surface pale yellowish-green, glabrous, except for a fringe of white tomentum on the margin, to densely covered with white, tangled stellate tomentum. Petals 6.5–10mmlong, 2–3mm wide, oblong, almost flator reflexed, pale yellow, becoming orange as they age. Stamens 38–46 in number, 7–11mmlong, exceeding the petals. Staminodes absent [10].	(widely planted in north-west Europe): **T. × europaea var. europaea (= 'Pallida') [10].**	but with small hairs forming a white tomentum on the edges; adaxial surface almost white with dense hairs covering the nectary. Petals 5.0mm long and 1.5mmwide, elliptical and clawed, clear yellow. Stamens 35–40, 7–8 mm long. Staminodes absent. Ovary 2.5 mm in diameter, densely covered in white tomentum; style initially 1–2 mm long and increasing to 5 mm, glabrous in some trees, in others with a dense covering of hairs which extends from the base up part or, as in the type, the whole of the style. °Stigmas divided into five short, rounded lobes: ***T. cordata*** subsp. ***Cordata*** [10]. Petals lanceolate, 4-5 mm long, stamens 5-6 mm long, staminodes absent, glabrous globose, abundantly hairy, bract 3-4 mm, glabrous, stamens five-lobed [37].
Bracts	5-12 cm long, 1-2 wide, glabrous [37]; 60–109 mm long and 11–22 mm wide, usually oblanceolate or occasionally oblong, sessile, or with a stalk to 16mmlong; apex semicircular or rounded, subacute; adaxial surface glabrous, except for a small patch of simple hairs in the axil of the peduncle, or with sparse fasciculate hairs; abaxial surface glabrous or with sparse to dense fasciculate hairs.	Hairy, basally [37]; oblong or lanceolate, sessile or stalked; °narrowed to both ends and expanded in the middle, very variable in length (to 100–130 mm) along the shoots (**T. × europaea var. europaea (= 'Pallida')**) [10]	4-8 cm long, 1-2 cm wide [37]; 40–87 mm long and 8–17 mm wide, elliptical to oblong, light yellowish-green, glabrous with a slender stalk, 6–24mm long [10].
Fruits	Spherical or oval 0.8-1 cm long, ribbed, hairy, brownish, with a thick incompressible shell [37], with distinct edges [34]; 9.1–11.9 mm long and 7.9–9.7 mm wide, spherical to obovoid, rarely ellipsoidal, normally pentagonal and ribbed when dry; wall 0.9–1.3mm thick, not easily broken, surface covered in dense white tomentum. °5–7 mm in diameter, ellipsoidal with prominent longitudinal ribs	Normally less than 8 mm diameter, spherical, broadly ovoid or obovoid, walls 0.4–0.8 mm thick, broken with difficulty [10]; spherical or on the contrary ovoid, ribbed, hairy, with a thick hard shell [37]; hard shell, short downy [34].	Spherical when sterile, ellipsoid to obovoid (6–7mm long, 3.5–4.2mm wide), ovoid with an asymmetric apex, walls 0.2–0.4 mm thick, fragile when dry [10]; oval 4-8 mm long, 5-7 mm in diameter, smooth or slightly ribbed, felty hairy, thin-walled, easily compressed shell, with 1-2 seeds [37]; spherical,

	when dry ***T. platyphyllos*** subsp***. Corinthiaca***<br>°9–11mm in diameter, spherical to broadly ovoid or obovoid (***T. platyphyllos*** subsp. ***Pseudorubra, T. platyphyllos*** subsp. ***Platyphyllos, T. platyphyllos*** subsp. ***Cordifolia***) [10].		pear-shaped, or slightly elongated [34].
	Distinguished by white bristly, long, visible hairs on young forks, leaves and even petioles [34].	Distinguished by parallel, sharp veins of the third-row [34].	

Table S2. Morphometric traits of observed *Tilia* sp. In the Pazaislis and Girionys parks.

	Nut form, fragility (+/-), ribs (+/-)	Brackt lower/uper side, Length (cm)	Leaf form Width × length (cm)	Veins: third-row (rised/horizontal or no), secondary (number)	Number of bud scales, Size of the outer scale (part from bud)	Uper part of leaf hairness	Lower part of leaf colour, hairness	Marginal teeth	Species
1	orbicular, +/-, -	-/-, 9-10	8x9, oblicue	+ 6-7	2-3 >1/2	no	Pale green; light brown stelate hairs in main veins axils	apiculate	T. europaea sub. Europaea
2	Orbicular, +, -/+	-/+-, 8-9	6x5, cordate	-, 6-7	2-3 3/5	no	Pale green; brown stelate hairs in veins axils	Subacute, apiculate, curved	T. cordata
3	Orbicular, +, +	-/-, 8-10	7x6, cordate	-, 6-7	2-3 ¾	no	Pale green; brown stelate hairs in veins axils	subacute	T. cordata
4	Ovate, +, -	-/-, 9-11	8x7 , cordate	-, 5-7	2-3 3/4	Yes - base	Pale green; brown stelate hairs in veins axils	Subacute, apiculate	T. cordata
5	Orbicular, +/-, +	-/+, 9-11	7x8, oblicue	-, 5-7	2 3/4	no	Pale green; light brown stelate hairs in base and some veins axils	apiculate,	T. europaea sub. Europaea
6	Ovate/ orbicular, +,-	-/-, 8-9	7x6,5, cordate	-/+, 6-8	2 ¾	no	Pale green; brown	subacute	T. cordata

	Nut form, fragility (+/-), ribs (+/-)	Brackt lower/uper side, Length (cm)	Leaf form Width × length (cm)	Veins: third-row (rised/horizontal or no), secondary (number)	Number of bud scales, Size of the outer scale (part from bud)	Uper part of leaf hairness	Lower part of leaf colour, hairness	Marginal teeth	Species
							stelate hairs in veins axils		
7	Ovate, +, +	-/-, 7-8	7x6, cordate	-/+, 6-8	2<br>¾	no	Pale green; brown stelate hairs in veins axils	subacute	T. cordata
8	Orbicular, -/+, -	-/-, 7-8	7x6, cordate	-/+, 6-8	2-3<br>1/3	no	Pale green; brown stelate hairs in veins axils	subacute	T. cordata
9	Orbicular, +, -	-/-+, 3,5-4	7x7, semi-cordate/ cordate	-, 6-7	3<br>1/2	no	Pale green; brown stelate hairs in veins axils	subacute, apiculate,	T. cordata
10	Orbicular, +, -	-/-, 7-8	7x6, cordate	-/+, 6-8	2-3<br>½	no	Pale green; brown stelate hairs in veins axils	subacute	T. cordata
11	Ovate, -/+, -	-/-+, 6-7	6x6, cordate	-, 5-7	3<br>>1/2	no	Pale green; brown stelate hairs in veins axils	Subacute, apiculate, curved	T. cordata
12	Orbicular, +, -	-/-, 5-6	6,5x6, cordate/ semi-cordate	-, 5-7	3<br><1/2	no	Pale green; brown stelate hairs in veins axils	subacute	T. cordata
13	Orbicular,+, -	-/-, 5-6	8x7,cordate	-/+, 6-8	2<br>1/1	no	Pale green; brown	apiculate, curved	T. cordata

	Nut form, fragility (+/-), ribs (+/-)	Brackt lower/uper side, Length (cm)	Leaf form Width × length (cm)	Veins: third-row (rised/horizontal or no), secondary (number)	Number of bud scales, Size of the outer scale (part from bud)	Uper part of leaf hairness	Lower part of leaf colour, hairness	Marginal teeth	Species
							stelate hairs in veins axils		
14	Ovate, +/-, -/+	-/-, 7-8	7x7, semi-cordate	-/+	2-3 <1/2	no	Pale green; brown stelate hairs in veins axils	subacute	T. europaea sub. Europaea
15	Ovate, +/-, -/+	-/-, 7-10	8x8, semy-cordate	-/+, 6-7	2-3 1/2	no	Pale green; brown stelate hairs in veins axils	apiculate	T. europaea sub. Europaea
16	Orbicular, +, -/+	-/-+, 6-9	6,5x6, cordate	-/+, 5-6	2 ¾	no	Pale green; brown stelate hairs in veins axils	Subacute, curved	T. cordata
17	Lanceolate, -/+, -	-/-, 5-6	5x5, slightly cordate	-, 5-7	2-3 <1/2	no	Pale green; brown stelate hairs in veins axils	Subacute, apiculate, curved,	T. cordata
18	Ovate, +, -	-/-, 6-7	6x6, slightly cordate/ semi-cordate	-/+,5-7	3 >1/2	no	Pale green; brown stelate hairs in veins axils	Subacute, apiculate, curved,	T. cordata
19	Orbiculate/ ovate, +, +/-	-/-, 8-10	7x6,5, cordate	-, 6-7	2-3 <1/2	no	Pale green; brown stelate hairs in veins axils	Subacute, apiculate, curved	T. cordata

	Nut form, fragility (+/-), ribs (+/-)	Brackt lower/uper side, Length (cm)	Leaf form Width × length (cm)	Veins: third-row (rised/horizontal or no), secondary (number)	Number of bud scales, Size of the outer scale (part from bud)	Uper part of leaf hairness	Lower part of leaf colour, hairness	Marginal teeth	Species
P1-P6	Orbiculate, -, +	-/+, 8-9	7×6, cordate	+, 7-8	3<br><1/2	no	Light brown, stelate hairs in veins axils	subacute	T. europaea
ML1	Obovate, +, -	-/-, 6-8	5,5×5, cordate	-, 5-6	2-3<br>1/2	no	Pale green; brown stelate hairs in veins axils	Subacute	T. cordata
ML2	Obovate, +, -	-/-, 5-7	6×5,5, cordate	-, 6-7	2<br>>1/2	no	Pale green; brown stelate hairs in veins axils	Subacute	T. cordata
ML3	Obovate, +, -	-/-, 6-7	5×6, cordate	-, 5-6	2<br>1/2	no	Pale green; brown stelate hairs in veins axils	Subacute, curve	T. cordata
DL1	Orbiculate, -,+	+/+, 7,5-8	8×7,5, cordate	+, 7-9	3, <=1/2	Yes, at base (rare), veins and petiole (dense)	White, dense fasciculate in axils, and medium density simple hairs in secondary and third row veins, and on petiolei	apiculate	T. platyphyllos
DL2	Orbiculate, -,+	+/+, 6-8	6,5×6,5, cordate	+, 7-9	3, <=1/2,	Yes, at base (rare), veins and petiole (dense)	White, dense fasciculate in axils, and medium density	apiculate	T. platyphyllos

	Nut form, fragility (+/-), ribs (+/-)	Brackt lower/uper side, Length (cm)	Leaf form Width × length (cm)	Veins: third-row (rised/horizontal or no), secondary (number)	Number of bud scales, Size of the outer scale (part from bud)	Uper part of leaf hairness	Lower part of leaf colour, hairness	Marginal teeth	Species
							simple hairs in secondary and third row veins, and on petiolei		
DL3	Orbiculate, -,+	+/+, 7-8	9×9,5, cordate	+, 7-8	3, <=1/2,	Yes, at base (rare), veins and petiole (dense)	White, dense fasciculate in axils, and medium density simple hairs in secondary and third row veins, and on petiolei	apiculate	T. platyphyllos

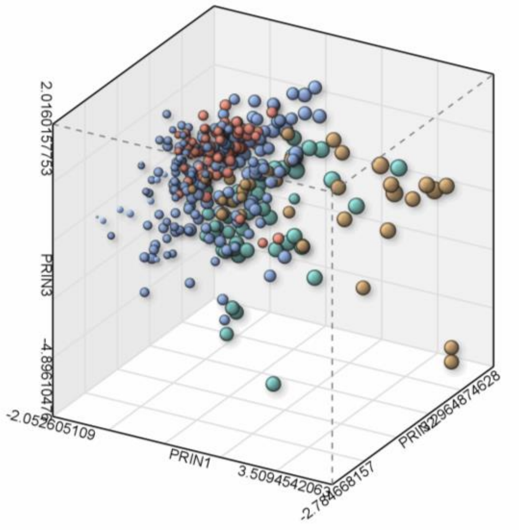
2.016015753
PRIN3
-2.052605109
PRIN1
3.509454206
PRIN2

A

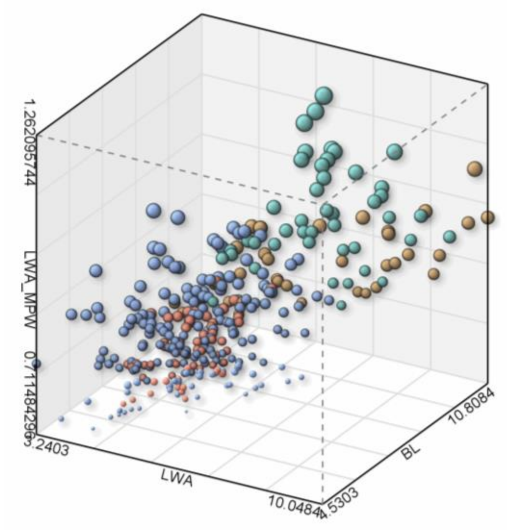
1.262095744
LWA_MPW
3.2403
LWA
10.0484
4.5303
BL
10.8084

B

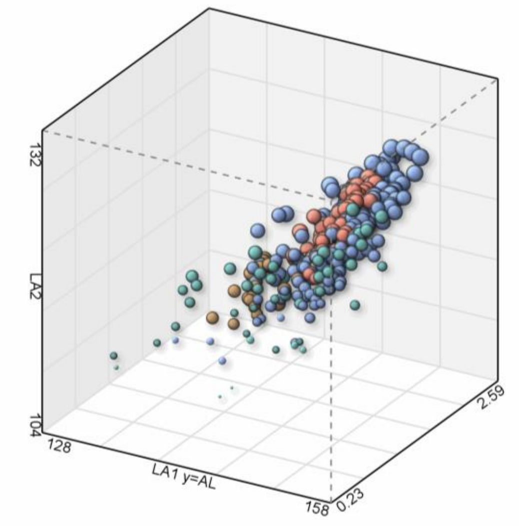
132
LA2
104
128
LA1 y=AL
158
0.23
2.59

C

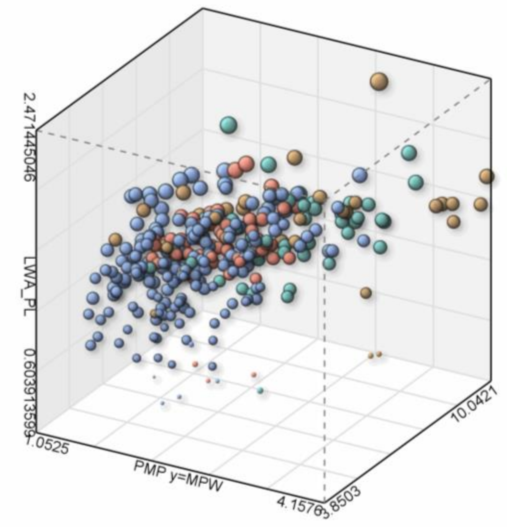
2.471445046
LWA_PL
1.0525
PMP y=MPW
10.0421

D

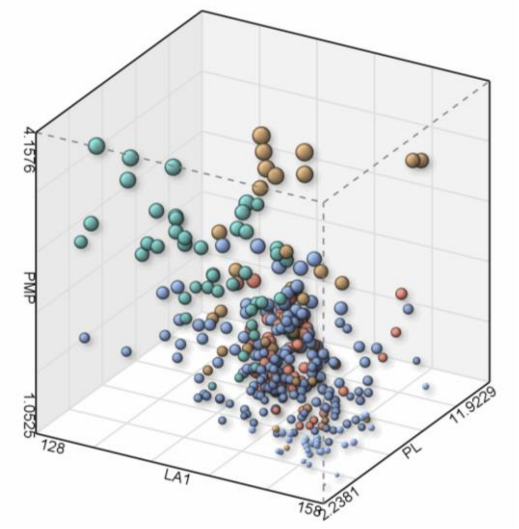
4.1576
PMP
1.0525
128
LA1
PL
11.9229

E

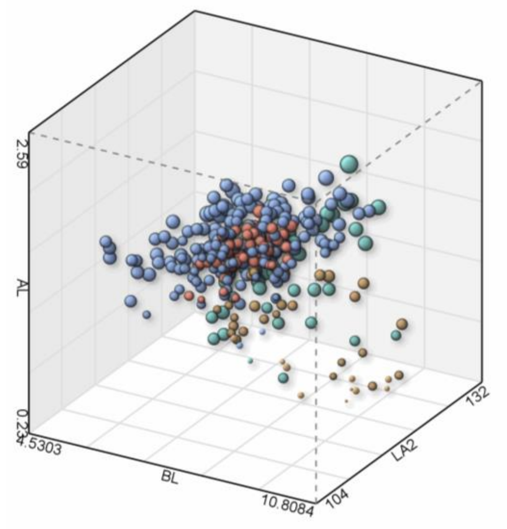
2.59
AL
4.5303
BL
10.8084
104
LA2
132

F

Fig S1. Different combinations of morphometric traits in 3D scatter plot [39]. *Tilia cordata* (- blue balls, *T.* × *europaea* – red balls, *T.* × *europaea* var. *europaea* ’Palinda‘ – green balls, *T. platyphyllos* – brown balls. Morphometric traits see in Table 1.
